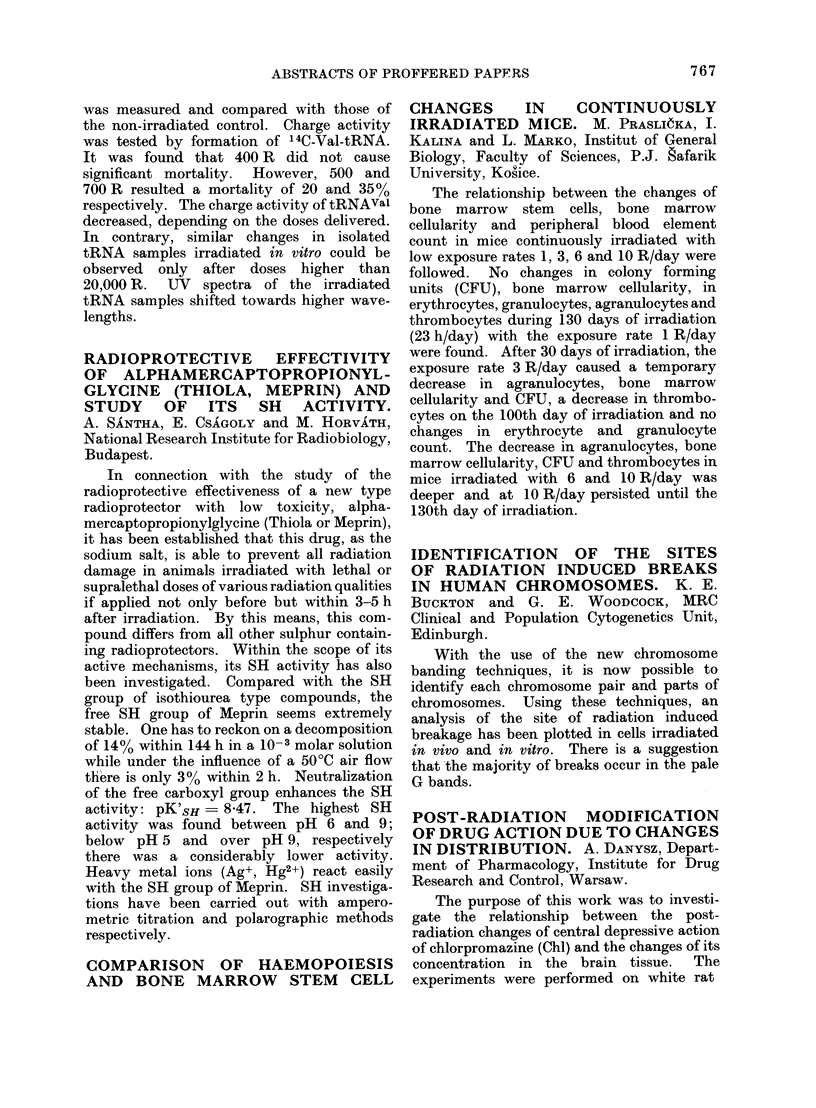# Identification of the Sites of Radiation Induced Breaks in Human Chromosomes

**Published:** 1975-12

**Authors:** K. E. Buckton, G. E. Woodcock


### IDENTIFICATION OF THE SITES OF RADIATION INDUCED BREAKS IN HUMAN CHROMOSOMES.

K. E. Buckton and G. E. Woodcock, MRC Clinical and Population Cytogenetics Unit, Edinburgh.

With the use of the new chromosome banding techniques, it is now possible to identify each chromosome pair and parts of chromosomes. Using these techniques, an analysis of the site of radiation induced breakage has been plotted in cells irradiated *in vivo* and *in vitro*. There is a suggestion that the majority of breaks occur in the pale G bands.